# The analysis of ramipril/ramiprilat concentration in human serum with liquid chromatography-tandem mass spectrometry – interpretation of high concentrations for the purposes of forensic toxicology

**DOI:** 10.1007/s12024-023-00621-6

**Published:** 2023-04-15

**Authors:** Marek Dziadosz, Wolfgang Rosenberger, Michael Klintschar, Jörg Teske

**Affiliations:** Institute of Legal Medicine, Hannover Medical School (MHH), Carl-Neuberg-Str. 1, 30625 Hannover, Germany

**Keywords:** Ramipril, Ramiprilat, Toxic concentration, Forensic toxicology, LC–MS/MS, Quantification

## Abstract

Ramipril is a popular angiotensin-converting enzyme inhibitor applied in the treatment of hypertension. Its therapeutic effect is oriented on the concentration of the active metabolite ramiprilat. The information about toxic drug levels is missing in the literature. Therefore, the aim of this work was an indication of possible toxic ranges based on the analysis of real samples with high ramiprilat concentrations. For these purposes, an appropriate analytical LC–MS/MS method was developed and validated according to forensic guidelines and applied in the routine. Most real samples targeted for ramipril/ramiprilat were associated with the typical therapeutic drug range of 1–40 ng/mL described in the literature. However, higher drug levels with ramiprilat concentrations above 100 ng/mL could also be observed infrequently in cases of driving under the influence of drugs or attempted suicides. To the best of the author’s knowledge, this is the first time antemortem ramipril and ramiprilat concentrations associated with driving under the influence of drugs and suicide attempts were discussed from a forensic point of view. The collected data enabled an indication of the ramiprilat toxic concentration range from about 600 ng/mL to at least 3500 ng/mL. The toxic concentration range discussed can be applied in the forensic practice as a reference for future cases.

## Introduction

Ramipril is a well-known angiotensin-converting enzyme (ACE) inhibitor adapted for clinical application in the treatment of hypertension since the late 1980s [1]. Since the ramipril metabolite ramiprilat is responsible for the major activity of the parent drug, both drug concentrations are important and of interest in different issues of forensic toxicology like driving under the influence of drugs or postmortem cases. Blood concentrations of drugs discussed were analysed in different studies. In detail, Mendes et al. reported an average ramipril/ramiprilat concentration of 13/9.8 ng/mL at 0.5/3.4 h after a single oral dose of 5 mg [2]. Based on Shionori et al. a single oral dose of 10 mg ramipril yielded an average ramipril/ramiprilat concentration of 18/4.7 ng/mL at 1.2/3.2 h [3]. Meyer et al. revealed that a dose of 20 mg ramipril resulted in an average ramipril/ramiprilat concentration of 52/34 ng/mL at 0.7/2.1 h [4]. The therapeutic effect of ramipril is estimated on the basis of the ramiprilat concentration expected in the range of 1–40 ng/mL [5]. No toxic/lethal ranges could be defined for the ACE inhibitor discussed.

In general, ramipril poisoning can be defined as rare. However, a monointoxication after an intended suicide with 20 ramipril tablets was described recently [6]. Additionally, a similar case with 100 tablets containing metoprolol and 20 tablets containing ramipril was described earlier by Wagner et al. [7]. No ramipril concentration values were published in the context of the described cases [6, 7]. Furthermore, postmortem concentration distribution was studied by Theofel et al. [8]. Ramiprilat concentration determined in femoral blood representing 33 cases was defined as comparable to therapeutic values expected for antemortem samples.

Ramipril/ramiprilat analyses are predominantly performed with conventional liquid chromatography-tandem mass spectrometry (LC–MS/MS) analytical methods. In this context, different sample preparation strategies based on protein precipitation, liquid–liquid extraction or solid-phase extraction were described in the literature [9–12]. Both the positive and negative electrospray ionisation (ESI) mode could be applied for these purposes.

Based on the literature, it can be stated that there is not enough information about the toxic/lethal concentration range of ramiprilat. Therefore, the aim of this work was (a) the development and validation of a simple protein precipitation based LC–MS/MS analytical method for the analysis of ramipril/ramiprilat in human serum and (b) analysis of real samples with an indication of possible toxic ranges.

## Materials and methods

### Chemicals and equipment

Chemicals and solvents used for the analyses were of analytical/LC–MS grade. Blank human serum was purchased from the blood bank of the Hannover Medical School and ramipril, ramipril-D3 and ramiprilat from Toronto Research Chemicals. Other materials used were purchased from J.T. Baker (methanol), Honeywell Riedel-de Haёn (water) and Merck (acetic acid, ammonium acetate).

Data acquisition was performed with a Sciex QTRAP 4500 LC–MS/MS System coupled with an ExionLC UHPLC system. Chromatographic separation was performed with an analytical column purchased from Phenomenex Luna 5 μm C18(2), 100 A, 150 mm × 2 mm. The analytical instruments used were operated with the analyst 1.7.2 software. Additionally, all calculations necessary for method validation were performed with the Valistat 2.0 software from Arvecon GmbH.

### Conditions

Two mobile phases with 10 mM ammonium acetate and 0.1% acetic acid (v/v) were applied for chromatographic separation and defined as A (H_2_O/methanol = 95/5, v/v) and B (H_2_O/methanol = 3/97, v/v). UHPLC was operated with a total flow rate of 0.5 mL/min, an oven temperature of 60 °C, a sample cooler temperature of 15 °C and with the following elution programme: (1) starting with 20% B, (2) ramping to 100% B between 0.00 and 2.50 min, (3) holding 100% B from 2.50 to 4.80 min, (4) reducing to 20% B between 4.80 and 5.00 min and finally (5) holding 20% B from 5.00 to 8.00 min. After 8 min, the system was equilibrated for the next run. The injection volume was 25 µL.

The LC–MS/MS system was operated with the multiple reaction monitoring (MRM) scan type and the following source/gas parameters of the positive electrospray ionisation (ESI): curtain gas (N_2_), 35 psi; collision gas, medium; ion spray voltage, 5500 V; temperature, 500 °C; ion source gas 1 (N_2_), 40 psi; ion source gas 2 (N_2_), 50 psi. Compound dependant parameters are summarised in Table 1.

**Table 1:** Detection parameters applied for ramipril, ramiprilat and the internal standard (ramipril-D3)

	**Q1 mass (Da)**	**Q3 mass (Da)**	**Time (ms)**	**DP (V)**	**EP (V)**	**CE (V)**	**CXP (V)**
Ramipril-T	417.2	234.1	50	101	10	31	2
Ramipril-Q	417.2	160.1	50	101	10	43	0
Ramiprilat-T	389.1	206.1	50	91	10	29	8
Ramiprilat-Q	389.1	156.0	50	91	10	29	0
Ramipril-D3-T	420.2	237.2	50	81	10	31	2
Ramipril-D3-Q	420.2	163.2	50	81	10	43	6

*DP* declustering potential, *EP* entrance potential, *CE* collision energy, *CXP* collision cell exit potential, *T* target, *Q* qualifier

### Extraction procedure

Protein precipitation was performed in a microcentrifuge tube. For these purposes, 10 µL of the internal standard (a methanolic solution with 100 ng/mL ramipril-D3) was mixed with 100 µL human serum and 300 µL methanol. After a shaking time of 15 min, the samples were centrifuged and the supernatant evaporated into dryness. The extract reconstitution was performed in a mobile phase mixture consisted of 80% A: 20% B (100 µL). Finally, extract filtration was followed by the injection into the LC–MS/MS system.

### Method validation

The analytical method was validated according to the guidelines of the German Society of Toxicological and Forensic Chemistry (GTFCh) [13]. Thus, the linearity of the calibration range was investigated by the analysis of the following calibration points: 1, 5, 10, 25, 50, 75 and 100 ng/mL for ramipril and ramiprilat. Each level was analysed six times. Additionally, the limit of detection (LOD) and quantification (LOQ) were calculated on the basis of calibration curves prepared in narrower concentration ranges 1–12.5 ng/mL. Selectivity of the method was investigated by the analysis of different lots of blank matrix performed 6 × with and 2 × without the internal standard. The matrix effect and recovery were evaluated by the strategy described by Matuszewski et al. [14]. For these purposes, a low (QC-L with 2.5 ng/mL ramipril/ramiprilat) and high (QC-H with 75 ng/mL ramipril/ramiprilat) quality control sample, with corresponding concentrations in the starting eluent and extracted blank matrix, were applied. The processed sample stability was investigated by the analysis of QC-L and -H samples performed at constant intervals during 3 h. Finally, both QC samples were applied for the calculation of method precision (intra- and inter-day precision) and accuracy. Appropriate, each QC sample was analysed twice a day during a time period of 8 days.

### Method application

The method developed was applied in the analysis of blood serum samples targeted for ramipril/ramiprilat provided by the police. Blood was collected in cases of both driving under the influence of drugs and attempted suicide.

## Results and discussion

### Method validation

**Fig. 1:**
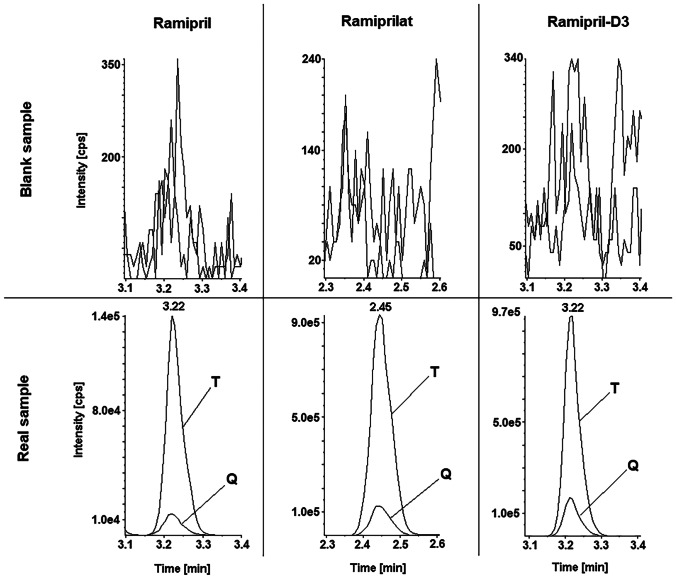
Chromatogram of a blank human serum and real sample with 1.6 ng/mL ramipril and 36 ng/mL ramiprilat (case 7 in Table 3); T, target, Q, qualifier

The results of the validation experiments are summarised in Table 2. Linearity of the calibration curve could be confirmed for both ramipril and ramiprilat in the range of 1–100 ng/mL. No interferences could be registered during selectivity experiments and the analysis of real samples performed. Typical chromatograms representing a blank human serum without internal standard and a positive real sample are presented in Fig. 1. The LOD and LOQ values enabled analyte analysis into the subtherapeutic concentration range and were comparable to other LC–MS/MS-based methods focused on the simultaneous ramipril/ramiprilat quantification [15, 16]. Additionally, method precision, accuracy and processed sample stability were in accepted ranges. As expected for a simple protein precipitation-based sample preparation, a good recovery could be achieved. The matrix effect observed for the drugs investigated could be defined as negligible and was in the range of 96–109% for ramipril (enhancement) and 93–94% for ramiprilat (suppression). In general, validation experiments demonstrated that the method developed can be applied for ramipril/ramiprilat analysis in human serum.

**Table 2:** Results of the validation experiments for ramipril and ramiprilat

	**LOQ**	**LOD**	**QC**	**Intra-day precision**	**Inter-day precision**	**Accuracy**		**Matrix effect**	**Recovery**	**Processed sample stability**
	**[ng/mL]**			**[%]**						
Ramipril	0.85	0.38	2.5	1.8	2.6	0.8	96		108	−22
			75	0.4	2.2	−6.4	109		104	−3
Ramiprilat	0.86	0.50	2.5	1.6	8.3	3.5	94		99	−22
			75	1.2	6.3	1.0	93		96	−4

### Method application

Since ramipril can be defined as a very popular drug applied for the treatment of hypertension, a lot of targeted quantifications are performed each year in our laboratory. The results of positive ramipril/ramiprilat cases summarised in Table 3 are selected examples of cases representing different analyte concentrations observed/expected in real samples.

**Table 3:** Examples of positive ramipril/ramiprilat quantifications performed in real samples

**Case number**	**Ramipril**	**Ramiprilat**
	**[ng/mL]**	
1	Negative	Approx. 0.86*
2	Negative	2.8
3	Negative	4.2
4	Negative	5.4
5	Negative	8.0
6	Negative	17
7	1.6	36
8	Negative	92
9	3.6	266**
10	7.5	562**
11	86	3560**

^*^Below the calibration range (approx. value); **above the calibration range (sample diluted)

Case 1 represents a subtherapeutic concentration of ramiprilat. Appropriately, the concentration calculated below the calibration range should be seen as an approximate value, whereas the cases 2–7 reflect typical ramiprilat concentrations observed in the majority of positive samples. They are characterised by a negative or low ramipril concentration and can be associated with a clinically intended therapeutic effect of the drug. Occasionally, atypical high ramiprilat concentrations could be observed (cases 8–11) with a moderate (cases 8 and 9) and strong concentration increase (cases 10 and 11).

## Discussion

In the analysis of postmortem ramiprilat concentrations, Theofel et al. already observed a concentration increase in a small number of cases [8]. However, these concentration increases were discussed as an effect of potential postmortem drug redistribution and were not further investigated.

In the evaluation of high ramiprilat concentrations, it is also important to note that in a study published by Heintz et al. practically no ramiprilat accumulation could be observed in human serum after 2 weeks [17]. On the basis of this information, high ramiprilat concentrations should be associated either with a drug abuse or with an inappropriate drug dosage. Based on the effect of ramipril, an intended drug abuse can be defined as contra-productive since an overdose can result in severe hypotension manifested usually within 6 h after the ingestion [18]. On the other site, a potential explanation of a ramipril misuse could be a neurological disease like dementia.

High ramiprilat concentrations can be explained by some health disorders and higher age. In general, according to the further literature, increased ramiprilat concentrations can be expected in the elderly and patients with renal impairment and heart failure [19]. In detail, even a ten times greater mean trough concentration was observed for patients with renal failure in comparison to healthy subjects [20]. On the other site, two separate studies demonstrated additionally that the maximal mean ramiprilat concentration in elderly (mean age ≥ 71 years) with normal renal function can be increased by 20% and 200% when compared to healthy young volunteers [19, 21, 22]. Although no negative effect on ramipril elimination could be observed for the patient groups mentioned, concentrations of the parent drug can also be potentiated in patients with hepatic impairment [19]. Since there are different explanations for increased ramiprilat concentrations, it seems probable that high drug levels observed in postmortem material can also be explained partly in this way [8]. Additionally, this aspect should also be taken into account in the interpretation of ramiprilat concentrations in antemortem samples when possible intoxications are considered.

High ramiprilat concentrations in cases 8 and 9 have an unknown genesis. However, an appropriate disease can usually be suspected after a detection of different drugs in human serum. Therefore, case 8 can be associated with a 71-year-old person. Bisoprolol, hydrochlorothiazide and blood alcohol (2.27‰) could be detected additionally to ramipril/ramiprilat, whereas in case 9 (50-year-old person), pregabalin, metformin, tilidine, ibuprofen and blood alcohol (2.30‰) could be detected as well. Only a high ramiprilat concentration with a relevant blood alcohol concentration could be defined as remarkable in the discussed cases 8 and 9. Under the assumption that prescribed drug doses were taken by car drivers, the high ramiprilat levels could be explained only by the age or/and different diseases [19–22]. Without the assumption of the intake of prescribed drug doses, high ramiprilat concentrations could also be discussed as a result of an intake of an excessive drug dose. Incorrect drug dosing can be performed, for example, under the effect of a high blood alcohol concentration.

Cases 10 and 11 are associated with the detection of ramipril/ramiprilat only. Case 10 is a confirmed suicide attempt with approximately 20 tablets with 5 mg ramipril and the person was described as heavily dazed, unsteady on his feet and with a wound on the wrist, whereas case 11 can be defined as a suspected suicide attempt with the drug (the information is strongly limited). Since case 10 can be defined as a ramipril/ramiprilat monointoxication with obvious adverse effects of the drug, the quantified ramiprilat concentration of 562 ng/mL represents a toxic drug concentration. Based on cases 10 and 11 (Table 3) a wide ramiprilat toxic concentration range can be assumed. A lethal ramiprilat concentration would occur when a problematic hypotension, resulted after a drug overdose, would not be treated in an appropriate way. Till now, no lethal ramipril/ramiprilat intoxication could be identified in our routine work.

The presented cases are associated with driving under the influence of drugs or attempted suicides. Therefore, the information was restricted to protocols provided by the police and no clinical data was available. Thus, high ramiprilat concentrations were interpreted on the basis of previous findings [19–22] and an assumption that therapeutic drug doses were taken [8, 9]. This fact can be seen as a limitation of the data evaluation presented since we do not assume as likely but cannot also fully exclude that these high ramiprilat blood serum concentrations could be a result of an inappropriate dosing. It should be also pointed out that reference drug ranges are very important in the forensic expertise. Since no toxic levels could be defined for ramiprilat, there is a lack of information about this popular drug [5]. Therefore, the toxic concentration range signalised in this paper from about 600 ng/mL to at least 3500 ng/mL can be defined as useful for the evaluation/interpretation of forensic cases in the future.

## Conclusions

An analytical LC–MS/MS-based method was developed for the parallel analysis of ramipril and its active metabolite ramiprilat after a simple and fast sample preparation strategy based on protein precipitation. Validation data confirmed the applicability for forensic toxicological quantifications. The method could be applied successfully for the targeted analysis of routine samples.

To the best of our knowledge, this is the first time antemortem ramipril and ramiprilat concentrations were discussed from the forensic point of view in the context of high and possible toxic levels. Until now, no toxic ramiprilat concentrations were specified in the literature for a forensic interpretation of an adverse drug effect. The presented data enables an indication of the toxic concentration range from about 600 ng/mL to at least 3500 ng/mL.

## Key points

1. Since no toxic concentration levels could be defined for ramipril/ramiprilat, the aim of this work was the analysis of real samples with an indication of possible toxic ranges. For these purposes, an LC–MS/MS analytical method based on a fast protein precipitation sample preparation strategy was developed and validated according to forensic guidelines.
2. The validation experiments and comparison with other methods published demonstrated the applicability of the LC–MS/MS quantification developed for the analysis of real samples.
3. The results of positive ramipril/ramiprilat cases revealed different analyte concentrations observed/expected in the routine with subtherapeutical, therapeutical and increased drug concentrations.
4. Based on the data presented an indication of the toxic concentration range from about 600 ng/mL to at least 3500 ng/mL was possible.

## Data Availability

Data are available upon request from the corresponding author.
